# The impact of piperine on the metabolic conditions of patients with NAFLD and early cirrhosis: a randomized double-blind controlled trial

**DOI:** 10.1038/s41598-024-51726-z

**Published:** 2024-01-11

**Authors:** Masoud Nouri-Vaskeh, Payam Hashemi, Naser Hataminia, Yalda Yazdani, Mahkameh Nasirian, Leila Alizadeh

**Affiliations:** 1 Immunology Research Center, Tabriz University of Medical Sciences, Tabriz, Iran; 2 Network of Immunity in Infection, Malignancy and Autoimmunity, Universal Scientific Education and Research Network, Tehran, Iran; 3 Faculty of Medicine, Tehran University of Medical Sciences, Tehran, Iran; 4 Liver and Gastrointestinal Diseases Research Center, Tabriz University of Medical Sciences, Tabriz, Iran

**Keywords:** Hepatology, Clinical trial design

## Abstract

Non-alcoholic fatty liver disease (NAFLD) is a metabolic dysfunction of the liver defined as an abnormal accumulation of fat within the liver without secondary triggers like alcohol consumption or viral hepatitis. Piperine, the bio-active ingredient of black pepper, can exert a significant function in treatment of individuals with NAFLDand early cirrhosis. We investigated the impact of piperine consumption with a duration of 12 weeks on patients with NAFLD and early cirrhosis compared toplacebo consumption. In a double-blind study, patients with NAFLD and early stage of cirrhosis were haphazardly distributed into case and control groups. They were prescribed a placebo and 5 mg of piperine for 12 weeks, respectively. The demographic and laboratory parameters of individuals were assessed as the baseline and after the duration of piperine intake. Piperine with a daily dosage of 5 mg could significantly decrease hepatic enzymes and glucose, and alleviate dyslipidemia in the case arm rather than the control arm. Moreover, HOMA levels and insulin resistance were reduced in case participants compared to the control counterparts. In the absence of approved medicinal intervention for patients with NAFLD, and regarding the favorable impact of piperine on NAFLD more studies on this subject are warranted.

## Introduction

Increased global proportions of unhealthy habits like sedentary lifestyle and Western diet lead to an enhanced prevalence of type 2 diabetes, obesity, fatty liver disease, and other metabolic disorders^1,2^. NAFLD, the most common type of chronic liver disease described with enhanced levels of lipid uptake within hepatocytes in the absent history of alcohol intake^3^. Decreased insulin sensitivity, hepatic inflammation, oxidative stress, and genetic risk factors can induce hepatic lipogenesis and NAFLD^4–6^. The inflammatory status of NAFLD may result in nonalcoholic steatohepatitis (NASH), cirrhosis, hepatocellular carcinoma, and liver failure^7,8^. This condition might be correlated with other chronic disorders including imbalanced Gut Microbiota, diabetes mellitus, obesity, cardiovascular disease, hyperlipidemia, chronic kidney disease, breast and colon carcinoma, and polycystic ovary syndrome^9,10^. However, there is no approved pharmacologic agent for management of patients with NAFLD^11^.

[(E, E) 1-[5-(1,3-benzodioxol-5-yl)-1-oxo-2, 4-pentadienyl] piperidine)], piperine, is a bio-active component of black pepper and exerts anti-oxidative^12^, anti-inflammatory, antihypertensive, neuroprotective^12^, and hepatoprotective^13^ effects. Piperine can also improve dyslipidemia possibly through activation of melanocortin-4 receptor within the central nervous system in a rat study^14^. The noted pharmacologic properties can make piperine a good fit for assessing its efficiency against chronic inflammatory diseases in clinical trials^15^. Beneficial role of piperine in hepatic lipid metabolism and NAFLD has also drawn attention of researchers^16–19^.

Here, we set out a study to evaluate the impact of piperine on NAFLD. We hypothesized that piperine can ameliorate the both severity and development of NAFLD.

## Methods

### Study design

This clinical intervention was implemented between August 2019 and July 2020 through a randomized double-blind placebo-controlled setting at a referral hospital. All experimental protocols were in accordance with the Declaration of Helsinki (2013). The principles of this study were accepted by Tabriz University of Medical Sciences Medical in the Ethics Committee (Registration Code: IRCT20180802040678N2, registered on 13/08/2019). Before enrollment, participants received detailed information about the clinical trial and possible complications of piperine and signed the informed consent.

### Study participants

The inclusion criteria encompassed grades 2, and 3 of NAFLD according to sonographic findings, with Alanine Transaminase (ALT) concentrations one and a half times higher than its upper limit of standard range. Grade 2 (moderate enhancement in echotexture of liver besides slightly impaired appearance of the portal vein wall and the diaphragm), and grade 3 (significant increase in hepatic echogenicity with poor or no visualization of portal vein wall, diaphragm, and posterior part of the right liver lobe) of steatosis was confirmed by ultrasound imaging^20^. The exclusion criteria comprised chronic liver disease, diabetes, malignancy, hereditary diseases that affect the liver such as iron and copper storage disorders, untreated hypothyroidism, autoimmune disease, pregnancy, lactation, alcoholic fatty liver disease, alcohol intake, history of hepatitis, using lipid-lowering, anti-inflammatory agents, and corticosteroids, methotrexate, tamoxifen and amiodarone, Bariatrics surgery in last year, intense diet to lose weight in the last 3 months, increased grade of the fatty liver based on ultra-sonographic findings during the study period, weight loss more than 10% during the study period, and patients who were not content with continuing the study.

### Sample size

The number of participants in this investigation was estimated at 54 individuals according to the data acquired by previous studies at a confidence level of 95%, with a power of 80%, and an effect size of 0.39 for Aspartate aminotransferase (AST) and ALT using GPower software (version 3.1.9.7). Regarding the estimated 20% attrition of participants, we confirmed 68 individuals as the sample size. The data of this study were exhibited as mean ± Standard Deviation.

### Randomization

Qualified patients were accidentally categorized into the case and control arms via Random Allocation Software. This software adjusts participants of every arm in terms of Model for End-stage Liver Disease (MELD) score, sex, and age. Produced random numbers by the software were preserved in a secure remote place. To confirm the blindness of the participant's distribution, it was performed with an independent investigator. In this study, all investigators and participants remained uninformed about the details throughout all steps of trial until gathering and analyzing the data.

### Intervention

Prior to the intervention, all participants were subjected to ordinary physical examinations. By using a Seca scale body weight was measured with an error of 0.1 kg (Kg). Height of individuals was determined by a standard tape with an error of 0.5 cm. Body mass index (BMI) of patients was obtained by dividing weight (Kg) by the height (meters) to the power of 2. Waist, hip, and neck circumferences of all enrolled individuals were determined with an error of 0.5 cm through their standard methods (in meters)^21^.

Blood sampling was conducted following 12 h of night fasting. 5 mL of venous blood was collected from each patient before and after the study interval. Laboratory data include Complete Blood Count (CBC), Albumin, total and direct Bilirubin, total cholesterol, AST, ALT, Alkaline Phosphatase (ALP), fecal calprotectin, Triglycerides (TG), total Cholesterol, High-Density Lipoprotein (HDL), Low-Density Lipoprotein (LDL), Homeostatic Model Assessment for Insulin Resistance (HOMA-IR), Fasting Blood Sugar (FBS), and Hemoglobin A1c (HbA1c).

**Figure 1:**
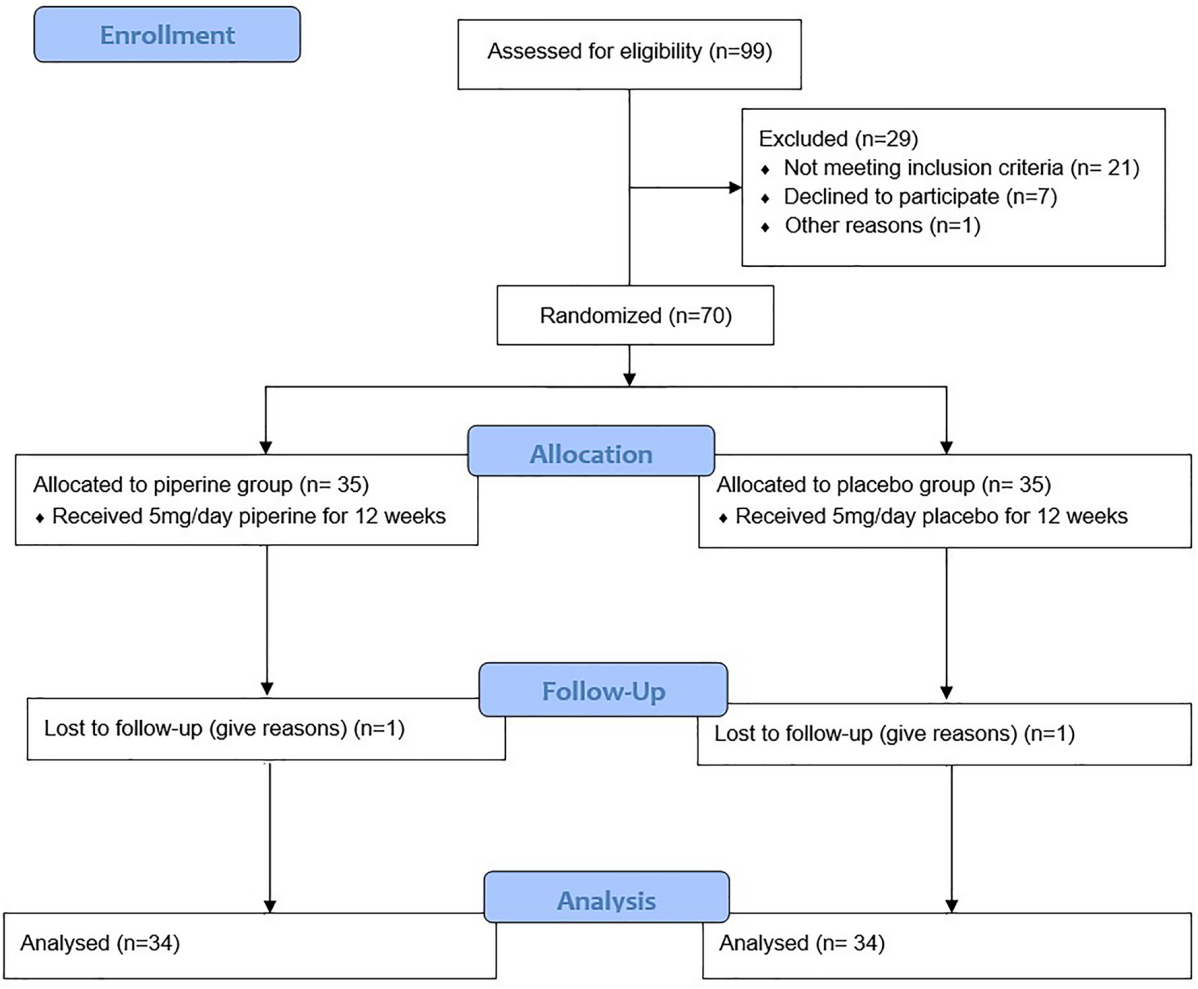
Study flow diagram.

A daily dose of 5 mg piperine capsule (piperine, Kimia Faravar Boali Co., Mashhad, Iran) was administrated to the members of case group (n = 34) for a duration of 12 weeks. Concurrently, patients in the control group (n = 34) were prescribed a placebo agent (placebo, Kimia Faravar Boali Co., Mashhad, Iran) for a duration of 12 weeks. More details included in Fig. 1.

Placebo capsules encompass a pharmacologically ineffective ingredient. The appearance of placebo capsules was indistinguishable from piperine capsules. Subjects were instructed to take the supplements with their launch. Nutritional intake and physical activity of enrolled individuals were as same as their routine plan during the trial interval. Enrolled participants were examined every week to evaluate the potential complications of piperine.

### Statistical analysis

By using version 18.0 of SPSS (SPSS Inc., Chicago, IL) the statistical analysis of this study was performed. Kolmogorov–Smirnov test was applied for assessing the normality of data distribution. Paired t-test was utilized to compare the difference between variables before and after the interval timeline. Between-groups analysis was completed using chi-square or independent sample t-test (according to the type of variables). To assess the differences between case and control groups Analysis of covariance (ANCOVA) was carried out before and after the study period. For assessment of intragroup and intergroup differences of categorical data Sign and Mann–Whitney U tests were done, respectively. The *P*-value amounts less than 0.05 was remarked as statistically meaningful results.

## Results

### Characteristics of study subjects

From 68 participants in the study, 33 individuals (48%) were men and 35 individuals (52%) were women. Baseline demographic indices including age, BMI, waist, and hip circumstance between the case and control groups were not significantly different (*P* value > 0.05). Detailed characteristics of demographic information have been shown in Table 1.

**Table 1:** Characteristics of study subjects.

Variable	Piperine group (n = 34)	Placebo group(n = 34)	*P* value
Age	41.76 ± 9.455	42.37 ± 11.282	0.815
Sex, n(%)			
Male	16 (47)	17 (50)	0.642
Female	18 (53)	17 (50)	
BMI	32.67 ± 4.52	31.14 ± 4.09	0.147
Weight	98.11 ± 16.61	97.41 ± 14.53	0.607
Waist	90.05 ± 4.63	91.70 ± 11.64	0.151
Hip	112.21 ± 11.94	108.83 ± 8.31	0.175

### Effects of piperine supplementation on biochemical variables

The biochemical characteristics of piperine and placebo groups have been detailed in Table 2. Except for the baseline levels of cholesterol and LDL, no other meaningful differences in baseline levels of biochemical parameters between case and control patients were detected.

**Table 2:** Biomedical characteristics of piperine and placebo groups before and after intervention.

Variable	Measurement period	Piperine group (n = 34) (mean ± standard deviation)	Placebo group (n = 34) (mean ± standard deviation)	*P*^a^
AST (IU/L)	Baseline	48.76 ± 17.21	54.80 ± 19.66	0.19
	After 12 weeks	35.50 ± 10.30	47.43 ± 18.02	0.002
	*P*^b^	< 0.001	< 0.001	
ALT(IU/L)	Baseline	81.13 ± 23.649	78.20 ± 26.032	0.633
	After 12 weeks	45.34 ± 9.367	67.80 ± 24.512	< 0.001
	*P*^b^	< 0.001	< 0.001	
Alkaline phosphatase	Baseline	222.79 ± 82.329	201.40 ± 69.141	0.249
	After 12 weeks	204.08 ± 62.853	191.03 ± 44.539	0.324
	*P*^b^	0.013	0.405	
Bilirubin (mg/dL)	Baseline	1.11 ± 0.38	1.09 ± 0.36	0.791
	After 12 weeks	1.19 ± 0.26	1.36 ± 0.83	0.428
	*P*^b^	< 0.001	0.659	0.659
Albumin (g/dL)	Baseline	4.71 ± 0.45	4.75 ± 0.41	0.732
	After 12 weeks	4.77 ± 0.33	4.50 ± 0.50	0.451
	*P*^b^	0.201	0.006	
Cholesterol (mg/dL)	Baseline	203.45 ± 44.46	225.57 ± 37.80	0.030
	After 12 weeks	171.55 ± 30.44	205.03 ± 37.21	< 0.001
	*P*^b^	< 0.001	< 0.001	
HDL (mg/dL)	Baseline	39.13 ± 6.55	42.13 ± 5.51	0.044
	After 12 weeks	43.03 ± 6.01	40.86 ± 4.90	0.109
	*P*^b^	< 0.001	0.311	
LDL (mg/dL)	Baseline	123.47 ± 25.46	134.20 ± 24.75	0.085
	After 12 weeks	105.74 ± 18.95	118.37 ± 28.79	0.043
	*P*^b^	< 0.001	< 0.001	
TG (mg/dL)	Baseline	234.24 ± 96.94	255.67 ± 175.03	0.551
	After 12 weeks	157.39 ± 41.29	214.23 ± 98.10	0.005
	*P*^b^	< 0.001	< 0.001	
FBS (mg/dL)	Baseline	105.26 ± 21.45	107.20 ± 19.97	0.702
	After 12 weeks	93.50 ± 14.05	102.50 ± 11.89	0.011
	*P*^b^	< 0.001	0.147	
HbA1c	Baseline	5.99 ± 0.91	6.10 ± 0.91	0.599
	After 12 weeks	5.81 ± 0.70	5.70 ± 0.56	0.767
	*P*^b^	< 0.001	0.383	
HOMA	Baseline	3.40 ± 0.71	3.39 ± 0.72	0.940
	After 12 weeks	2.89 ± 0.84	3.15 ± 0.49	0.596
	*P*^b^	0.000	0.258	
GGT (U/L)	Baseline	53.37 ± 24.48	57.20 ± 25.11	0.530
	After 12 weeks	54.72 ± 15.23	75.40 ± 26.95	0.042
	*P*^b^	0.004	0.570	

*P* value < 0.05 was considered significant.

Abbreviations: ALT, alanine aminotransferase; AST, aspartate aminotransferase; HDL, high-density lipoprotein; LDL, low-density lipoprotein; TG, triglycerides; FBS, fasting blood sugar; Hba1c, hemoglobin A1c; HOMA, homeostatic model assessment; GGT, gamma-glutamyl transferase.

^a^*P* values represent the comparison between arms (independent sample t-test at baseline and analysis of covariance test following 12 weeks of study interval.

^b^*P* values represent comparison within arms (paired t-test).

After the trial interval, concentrations of ALT and AST in the case group were statistically decreased compared with the control group with a *P* value < 0.001 and a *P* value of 0.002, respectively. Levels of AST and ALT within the both case and control participants were statistically reduced after the study period (both with *P* value < 0.001).

After the study interval, levels of ALP, bilirubin, and albumin were not significantly reduced in case group in comparison to control group (*P* value = 0.324, 0.428, 0.451, respectively). Serum levels of ALP and bilirubin significantly declined within case arm (*P* value = 0.013 and *P* value < 0.001, respectively) in comparison to control arm after the study interval (*P* value = 0.405 and 0.659, respectively). Conversely, albumin levels were not significantly decreased after the intervention within case group (*P* value = 0.201) in comparison to the control counterparts (*P* value < 0.001).

Serum concentrations of cholesterol, LDL, and TG were significantly reduced after the timeline of intervention in participants of case arm in comparison to the participants of control arm (*P* value < 0.001, *P* value = 0.043, *P* value = 0.005, respectively). In contrast, HDL concentrations were not significantly lessened in case group in comparison to control group following the study timeline (*P* value = 0.109). Serum concentrations of cholesterol, LDL, and TG were significantly decreased within case arm and control arm after the intervention (all with *P* value < 0.001). HDL concentrations were significantly decreased within the case group (*P* value < 0.001) in comparison to control group after the intervention (*P* value = 0.311).

Serum levels of gamma-glutamyltransferase (GGT) were statistically declined in the patients of case arm in comparison to patients of control arm after the intervention (*P* value = 0.042). Serum levels of GGT were significantly decreased within the control group (*P* value = 0.004) rather than the control group after the intervention (*P* value = 0.570).

Serum levels of FBS significantly declined in case arm in comparison to control arm after the trial timeline (*P* value = 0.011). FBS and HbA1c levels were significantly decreased within case individuals (both with *P* value < 0.001) in comparison to placebo individuals (*P* value = 0.147, 0.383, respectively) after the intervention.

Levels of HOMA were not significantly declined in patients of case group in comparison to control patients (*P* value = 0.596) after the intervention. Levels of HOMA were significantly decreased within case group (*P* value < 0.001) in comparison to control group (*P* value = 0.258) after the intervention.

The mean levels of platelets at baseline and after intervention were 180.92 ± 27.79 (*10^3^) and 195.42 ± 40.21 (*10^3^) within the case group, respectively. There was no report of thrombocytopenia among patients in participants of case group (which receive piperine).

### Safety of piperine supplementation

In order to minimize side effects risk, the daily dosage of piperine administration was 5 mg which is considerably less than its maximum allowed daily dosage and the previous observation^22^. Patients did not describe any complications during the prescription of piperine or placebo, which established safety of piperine in the present study. We did not detect any side effects related to piperine consumption during our clinical trial (N = 0).

## Discussion

The desirable influence of Mediterranean diet and antioxidant intake in amelioration of lipid profile, anthropometric parameters, and hepatic lipid absorption in overweight patients with NAFLD have been demonstrated^23^. Therapeutic effects of piperine on chronic diseases such as metabolic syndrome, obesity, diabetes, hepatic steatosis, arthritis, Alzheimer’s disease, Parkinson's disease, hypertension, malignancies, cardiovascular diseases, cerebral stroke, chronic renal diseases, and inflammatory diseases have also drawn the attention of medical researchers^16,24–30^. Along the way, some investigations have evaluated the impact of curcumin and a combination of piperine and curcumin on patients with NAFLD^31–35^. However, no clinical trial has been conducted on the action of piperine alone in management of patients with NAFLD yet to now.

The consequences of this clinical trial have shown the helpful function of piperine in biochemical variables of patients with NAFLD and early cirrhosis which fortifies the outcomes of other observations^33,36–38^. Piperine could significantly reduce hepatic enzymes, and ameliorate lipid and glucose metabolisms in this study. Piperine could also reduce insulin resistance and HOMA levels within case group compared with control group.

Piperine exerts its favorable action in insulin resistance and hepatic steatosis through impairment of Lxr-α, Srebp1c, CD36, Chrebp-α, and Fas in high-fat diet mice model^39^. Piperine can also reduce the levels of hepatic lipogenesis via inducing the transporter protein of ATP binding cassette sgm8 (Abcg8) and hepatic scavenger receptor b1 (Sr-b1) in small intestine of high-fat diet mice^40^. Du et al., have demonstrated the efficient role of piperine in reducing the mass of visceral adipose tissue rather than subcutaneous adipose tissue in mice model. They also detected the desirable role of piperine in alleviating glucose levels, and dyslipidemia, and downregulating the m-RNA expression of Sfrp5, MEST, and PTRF/Cavin1^41^. Moreover, researchers have discovered the anti-fibrotic properties of piperine within liver, pancreas, heart, skin, and submucosal tissues via different mechanisms in animal studies^42–46^. In addition, the desirable role of piperine against hepatocellular carcinoma has been reported^47–49^.

Metabolic effects of piperine have also been investigated in other studies^29,36,50,51^. In animal models of diabetes and obesity, piperine can increase the metabolic rate by increasing the ATPase activity of myosin chains within skeletal muscles^52^. Piperine can also improve insulin resistance through its immunomodulatory effects such as declining in white blood cell count, lipopolysaccharide, galectin-3, interleukin-1β, macrophages with M1-like polarization, and levels of mRNA of pro-inflammatory cytokines in fat tissue of mice model with diabetes^53^. This alkaloid can inhibit the non-enzymatic glycation of albumin and subsequent erythrocyte hemolysis. Piperine can also protect the erythrocyte membrane, and preserve its antioxidant capacity in the in-vitro environment^54^. Piperine and its derivatives significantly alleviate the conditions of diabetic nephropathy by decreasing the signaling of TNF-α, NLRP3 inflammasome, NF-κB, and IL-1β in a diabetic rat model^55^. Piperine exerts its protective role against hyperglycemia via stimulation of the Camkk/Ampk signaling pathway (in a reactive oxygen species-dependent manner) and thereby enhances the levels of glucose absorption in rat's skeletal muscle^56^. In alloxan-induced diabetic mice, a dosage of 20mg/Kg piperine administration can also lower glucose levels^57^. Moreover, in streptozotocin-induced diabetic mice, piperine can increase pancreatic superoxide dismutase and decrease malondialdehyde (MDA) leading to amelioration of oxidative stress within the β-cells of the pancreas and inhibition of apoptosis of these cells^58^.

He Jianlin and his colleagues reported the beneficial influence of piperine in fatty liver disease, peri-renal thickness of adipose tissue, and gastrointestinal microbiota in mice model with high-fat diet^50^. Action of piperine against insulin resistance and hepatic steatosis has been demonstrated in rat model with high-fat diet-induced obesity through the impairment of adiponectin-Ampk and PI3K-Akt signaling^51^. Piperine has also demonstrated favorable function by inducing Ucp1 expression and Ampk-p38-Mapk signaling pathway leading to production of lactic acid within skeletal muscle^59^. Another investigation has shown the regulatory action of piperine in metabolism of carbohydrate, lipid, and redox pathways within skeletal muscle of mice during endurance exercise^60^. Piperine can also ameliorate lipid metabolism, circadian rhythm, oxidative stress, and mitochondrial dysfunction in HepG2 cell line by inducing AMPK/AKT-mTOR and SREBP-1c/PPARγ pathways^61^.

Although the initial results are encouraging to show the very preliminary trend in this therapeutic area, as the next step the effect of the Piperine should be ascertained by randomized double-blind placebo-controlled, sample size driven studies with imaging techniques like fibroscan as endpoints for a longer duration like 6/12 months. The basic and fundamental limitation of the study is diagnosis of NAFLD for enrollment of subjects, therefore future trials should focus on the three components of NAFLD with well established methodologies such as fibrosacn or biopsy: Steatosis, inflammation, and fibrosis. The imorptant limitations of the study is that sonographic assessment of Liver was performed only at baseline (that covers only steatosis part of NAFLD), hence change could not be evaluated for steatosis after treatment with Piperine.

We could get trend that Piperine decreases hepatic enzymes including AST, and ALT, and improves lipid and glucose metabolism in individuals with early stages of NAFLD in this small study, however with lack of robust diagnosis and end points. Due to the lack of an approved pharmacologic agent for management of NAFLD and cirrhosis, further studies about the hepatic effects of Piperine should be encouraged.

## Data Availability

The datasets used and analyzed during the current study are available from the corresponding author upon reasonable request.
